# An empirical investigation on the correlation between solar cell cracks and hotspots

**DOI:** 10.1038/s41598-021-03498-z

**Published:** 2021-12-14

**Authors:** Mahmoud Dhimish, Pavlos I. Lazaridis

**Affiliations:** 1 Department of Electronic Engineering, University of York, Heslington, YO10 5DD York UK; 2 Department of Engineering and Technology, University of Huddersfield, Huddersfield, HD1 3DH UK

**Keywords:** Energy science and technology, Engineering, Materials science

## Abstract

In recent years, solar cell cracks have been a topic of interest to industry because of their impact on performance deterioration. Therefore, in this work, we investigate the correlation of four crack modes and their effects on the temperature of the solar cell, well known as hotspot. We divided the crack modes to crack free (mode 1), micro-crack (mode 2), shaded area (mode 3), and breakdown (mode 4). Using a dataset of 12 different solar cell samples, we have found that there are no hotspots detected for a solar cell affected by modes 1 or 2. However, we discovered that the solar cell is likely to have hotspots if affected by crack mode 3 or 4, with an expected increase in the temperature from 25$^\circ$C to 100$^\circ$C. Additionally, we have noticed that an increase in the shading ratio in solar cells can cause severe hotspots. For this reason, we observed that the worst-case scenario for a hotspot to develop is at shading ratios of 40% to 60%, with an identified increase in the cell temperature from 25$^\circ$C to 105$^\circ$C.

## Introduction

**Figure 1:**
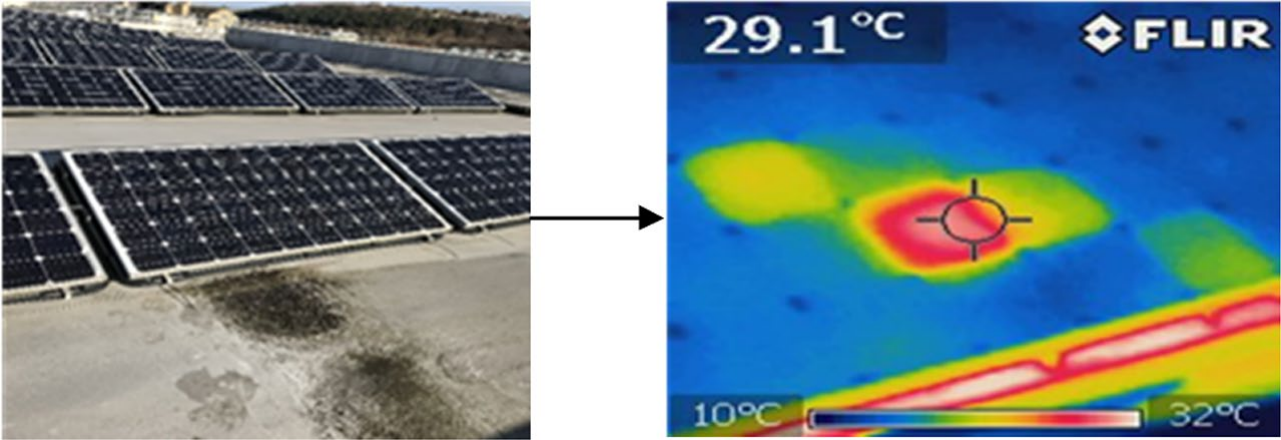
The physical and thermal image of a PV module is affected by hotspots; the thermal image is captured using a FLIR i5 thermal imaging camera, which has a thermal sensitivity of ± 1$^\circ$C.

To aid the transition to green energy, there has been a considerable demand to combine more photovoltaic (PV) systems into the electric grid to support the growth of renewable energy sources. As such, PV reliability and durability receives great interest from the industry as there are currently various mismatching conditions affecting these installations^1–3^. One of the challenges in today's PV modules is the well-known phenomenon called hotspots^4^. A hotspot is a localized heat source that can be present in part(s) of the PV module, leading to locally increased temperature in the solar cells. An example of a PV module affected by hotspots is shown in Fig. 1.

There are currently undergoing investigations on how PV hotspots occur in PV modules. Some researchers suggest that hotspots are present due to the rapid change in the night-to-day temperature^5^, particularly in deserts and coastal areas^6^. However, there is no complete demonstration of how hotspots develop, especially for new PV modules that are not affected by any means of cracks or physical damages. For example, Dhimish et al.^7^ observed that hotspots are likely to develop in cracked solar cells, and they show that a complete hotspot string within a PV module could lead to a 25% loss in output power. At the same time, the temperature could also increase by up to 65$^\circ$C. In this study, the examined PV sub-strings were operated under indoors conditions.

There were some attempts to explain how cracks could lead to hotspots^8,9^, yet this has not been fully reported in the literature. It is worth noting here that cracks are generally presented as one mode; however, some recent studies^10–12^, have explained that cracks could be classified into different modes, including micro-crack and breakdown regions. This topic has been of great interest to the industry because solar cell cracks are proven to affect the output power yield and several studies evidence^13,14^ that this could lead to a significant drop in the solar cells' other electrical parameters, such as the open-circuit voltage, short circuit current, and the fill factor. The problem occurs when the crack area is electrically disconnected, limiting the maximum output current generation and, as a result, leading to an electrical mismatching condition.

The mitigation of solar cell cracks has not been yet discovered. However, several studies explain how to mitigate solar cell hotspots. The old-fashioned approach^15,16^ is to connect the PV sub-strings in parallel with a bypass diode. This technique allows the current to have a different path and allows the unshaded solar cells to generate more current. Recent techniques such as^17–20^ have proposed the same principle, although adopting a metal–oxide–semiconductor field-effect transistor (MOSFET) in series with the cracked/hotspot PV string. The technique allows the MOSFET to regulate the current. It was confirmed that these techniques improve the output power of affected PV modules. However, their main limitations are, (i) the requirement of additional power supply to control the MOSFET switching, (ii) the internal power electronics circuit dissipating power, and (iii) these techniques are complicated to configure with many solar cells; customarily, they connect along with full-scale PV modules rather than individual solar cells.

The main contributions of this paper are, (i) an investigation of the correlation of solar cell crack modes and the presence of hotspots; four different crack modes were identified based on captured electroluminescent (EL) images of the examined solar cells, (ii) a mapping of the correlation between the cracks modes based on empirical results of measured output power, and (iii) a better understanding of the relationship between the shading ratio in the original EL image and the presence of hotspots.

## Materials and methods

### Test samples

The examined solar cell samples have been dismounted from 22 series-connected PV modules operating in the field for five years, and all were in the same PV site located near Leeds city in the UK. The PV manufacturer carefully managed this process to ensure no extra cracking or further damage was induced to the cells. The solar cells are polycrystalline silicon (poly-Si) with a peak power of 3.66 W at standard test conditions (STC), where the solar irradiance is 1000 W/m^2^ and cell temperature 25$^\circ$C. Each cell has a 3.2 mm glass superstrate, an ethylene–vinyl acetate (EVA) encapsulant^21^, and AAA backsheet substrate^22^.

### Scientific methods and techniques

**Figure 2:**
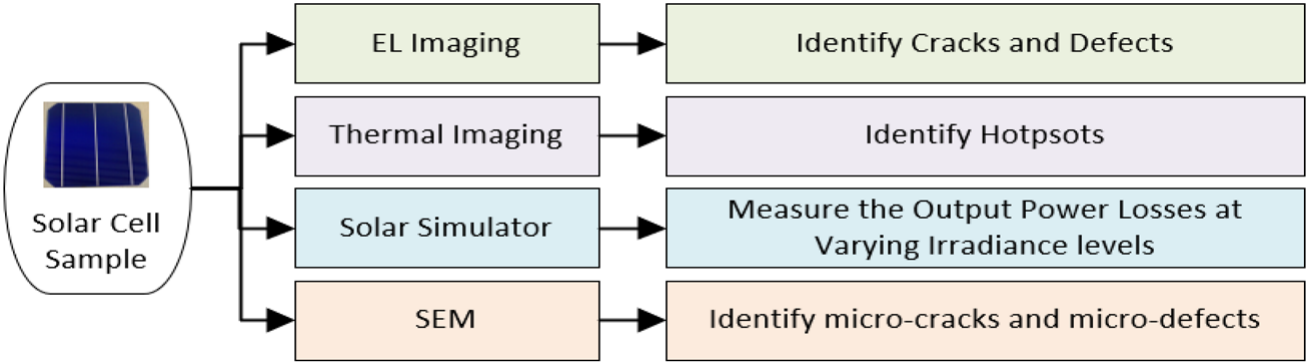
Detection methods used in this study.

The scientific methods/techniques to study the solar cell samples are summarised in Fig. 2. EL imaging is used to identify the cracks and defects in the cells^23,24^, whereas thermal imaging is applied to determine whether the cell sample has a hotspot. The solar cell samples have been tested under the sun simulator at STC conditions, where the cell was illuminated at 1000 W/m^2^ light intensity. The temperature of the cell was measured from the rear (non-illuminated) side using a thermal camera. For every sample, the temperature was left to develop for 10 min before measurement. This allowed sufficient time for the hotspots, if any, to reach a steady state with minimal fluctuations observed. And finally, to observe any micro-level fractures and defects in the cells, we have used scanning electron microscopy (SEM).

### Electroluminescent, thermal imaging, solar simulator, and SEM

**Figure 3:**
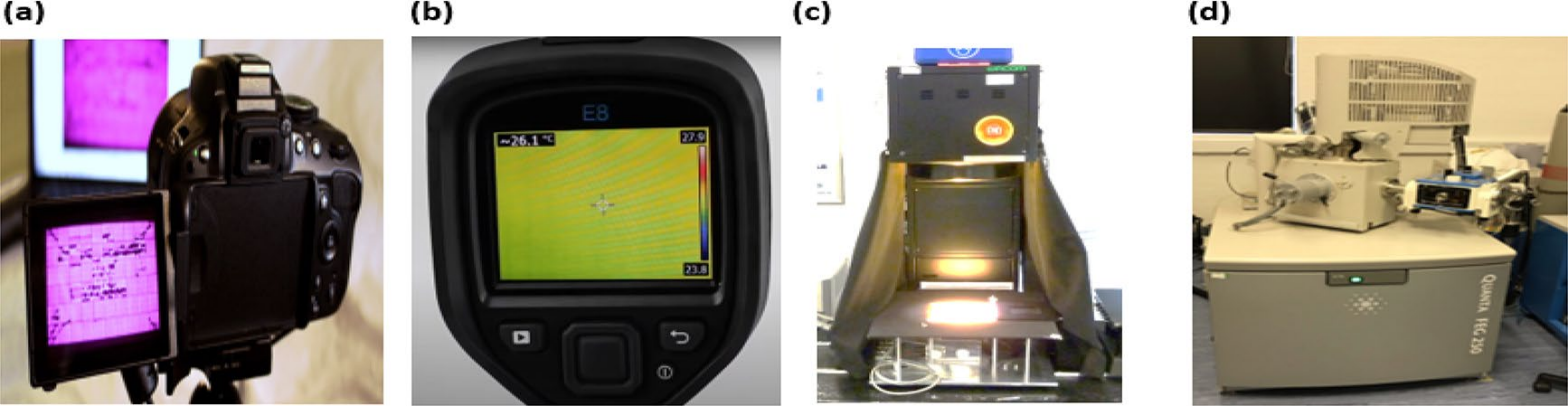
Experimental setup (**a**) EL imaging camera, (**b**) thermal camera, (**c**) solar simulator, and (**d**) scanning electron microscope.

The EL images of the solar cell samples are taken using the EL camera shown in Fig. 3a. The camera comprises a digital single-lens reflex with a high resolution of 6 k $\times$ 4 k pixels. The infrared cut-off filter is removed and calibrated to allow sensitivity to the electroluminescence picture with a peak wavelength of 1150 nm. The camera has an adjustable lens of 18–55 mm. The accuracy of the EL image depends on the distance between the EL camera and the solar cell. In our experiment, the distance was 2.4 m, which produces the highest quality EL images.

To capture the thermal image of the solar cells, a FLIR E8-XT thermal imaging camera is used (Fig. 3b). The thermal camera has a thermal resolution of 320 × 240-pixel and 0.05$^\circ$C thermal sensitivity. The camera was placed 2.4 m apart from the solar cells to detect the entire area of the inspected cells.

Finally, to investigate the performance of the solar cells under varied solar irradiance (0–1000 W/m^2^), a steady-state solar simulator is used, presented in Fig. 3c. The solar simulator is a class A + spectral match, IEC60904-9, with a ± 2% illumination sensitivity. In addition, we have used the Quanta FEC250 SEM, Fig. 3d, to inspect the microstructure of the examined solar cells. The SEM has an adjustable magnification of up to 2,000,000x, and the resolution of the digital output image can be as high as 4096 × 3536.

## Results

### EL imaging

**Figure 4:**
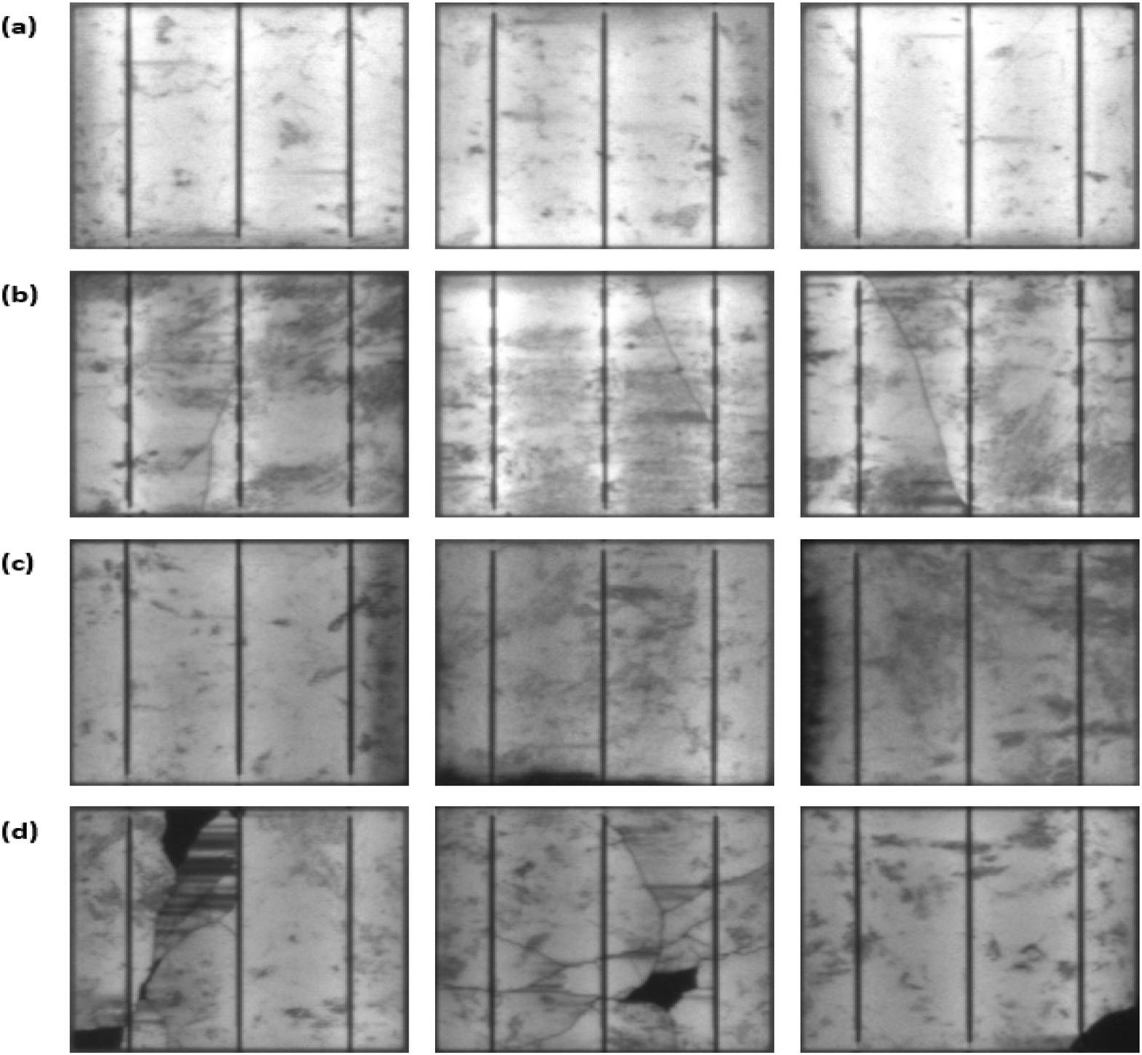
EL image of the examined solar cell samples (**a**) mode 1, (**b**) mode 2, (**c**) mode 3, and (**d**) mode 4.

Every solar cell was subjected to EL imaging to inspect the fracture and the crack mode. In this study, we have investigated four different modes: (i) crack free (mode 1), (ii) micro-crack (mode 2), (iii) shade area (mode 3), and (iv) breakdown (mode 4). For every mode, three different solar cell samples were selected, all presented in Fig. 4. Here the cracks were classified into four different modes. In contrast, in previous research^25,26^, solar cell cracks were distinguished by either a singular crack mode, named micro-crack or µcrack. We find our classification is significant because not every crack mode can lead to hotspots or a substantial drop in the output power.

Cracks formed in the solar cells for various reasons, including defective manual soldering, improper installation of the PV modules in the PV site, transportation, and unavoidable materials defects. The cracks themselves cannot be mitigated/alleviated, and they are likely to grow as the solar cells continue to be exposed to solar irradiance and to fluctuations in temperature.

### Thermal performance of the tested solar cell samples

**Figure 5:**
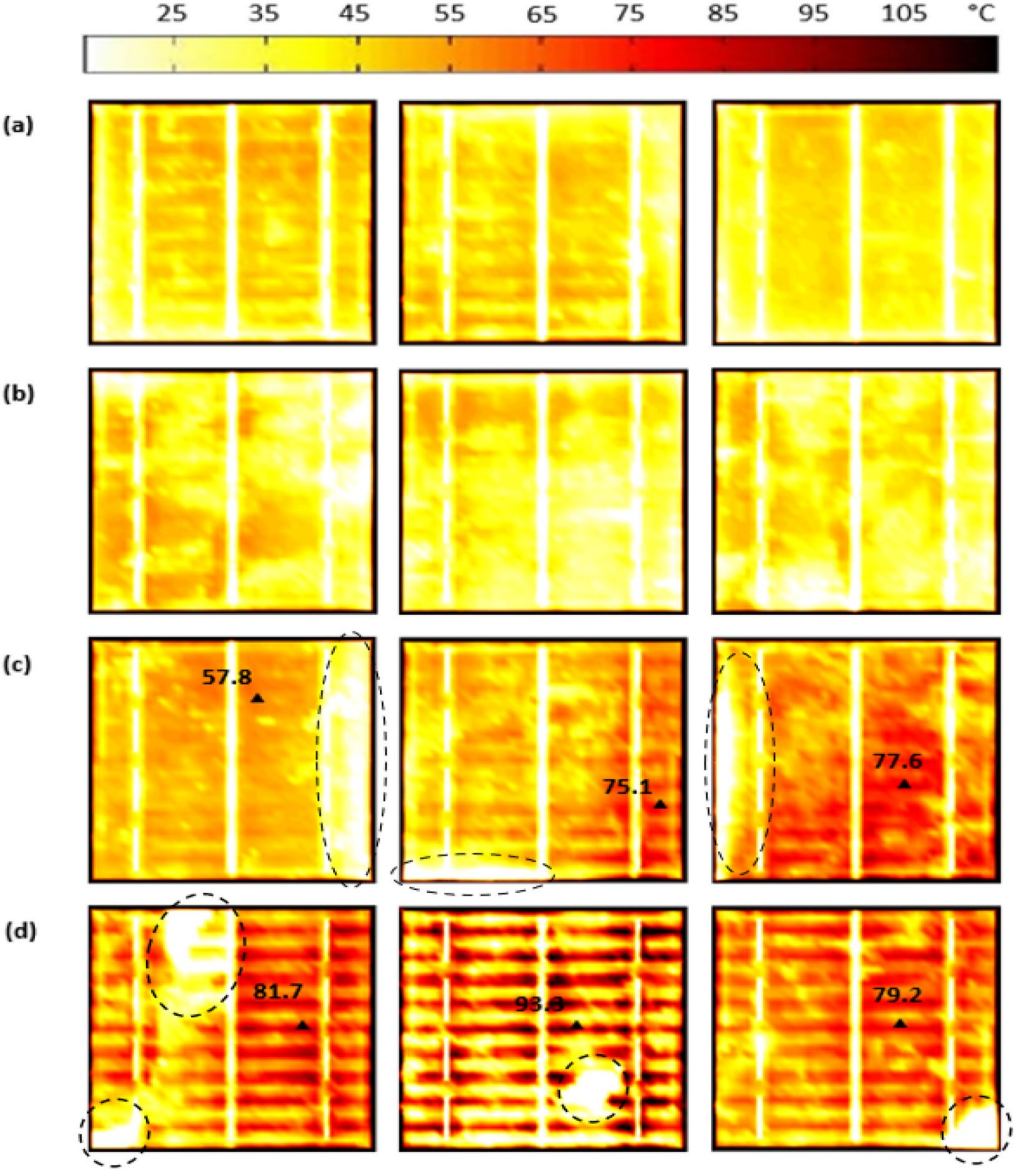
Thermal image of the examined solar cell samples under STC condition (**a**) mode 1, (**b**) mode 2, (**c**) mode 3, and (**d**) mode 4.

The results of the thermal testing are shown in Fig. 5. According to Figs. 5a,b a uniform distribution of the heat across the solar cell surface was detected. Accordingly, the temperature is approximately at the standard testing condition, 25$^\circ$C. It is noticed that no hotspots are exposed to the solar cell samples with crack-free (mode 1) and micro-cracks (mode 2). Furthermore, for crack modes 1 and 2, there was no increase in the cell's temperature, which confirms that crack-free or micro-cracks do not change the temperature of the cells and are unlikely to develop a hotspot.

In contradiction, if we consider Fig. 5c, mode 3, the shade in the cells causes no increase in the temperature, labelled in dashed circles. The shade will create an uneven distribution of the current flow in the busbars; this would generate stress in the cell and then cause an increase in the temperature. We can observe that the samples are affected by the rise to the surface temperature up to 77.6$^\circ$C. Hence, if these samples were under field operation, hotspots would be expected. We yet do not fully understand the correlation between the shading ratio in the original EL image and the presence of hotspots; this case will be detailed later in the article.

According to mode 4, Fig. 5d, the breakdown regions in the solar cells have no increase in the temperature, labelled in dashed circles. This is explained by the fact that there is no contact in the inner and outer layers of the cells. Hence, no photo illumination is observed. In contrast, the cells are now being impacted by an increase in the surface temperature (of up to 93.3$^\circ$C) due to these breakdown regions. The same explanation is valid as in crack mode 3, where breakdown regions affect parts of the solar cells, which would create an uneven distribution of the current flow in the busbars, leading to this significant increase in the cell surface temperature. A similar observation was made in the recent study by Akram *et* al.^27^, where they discovered that a hotspot could lead to an increase in the cell surface temperature of up to 110$^\circ$C.

The results of the development of the hotspots in crack modes 3 and 4 lead us to explain why in some commercial PV systems, some modules do not develop hotspots even though they appear to have snail trails^28^ or cracks. Accordingly, for a solar cell to exhibit severe hotspots, it certainly depends on the cracking mode and how long this crack has been settled in the solar cell sample.

### Output power measurements

**Figure 6:**
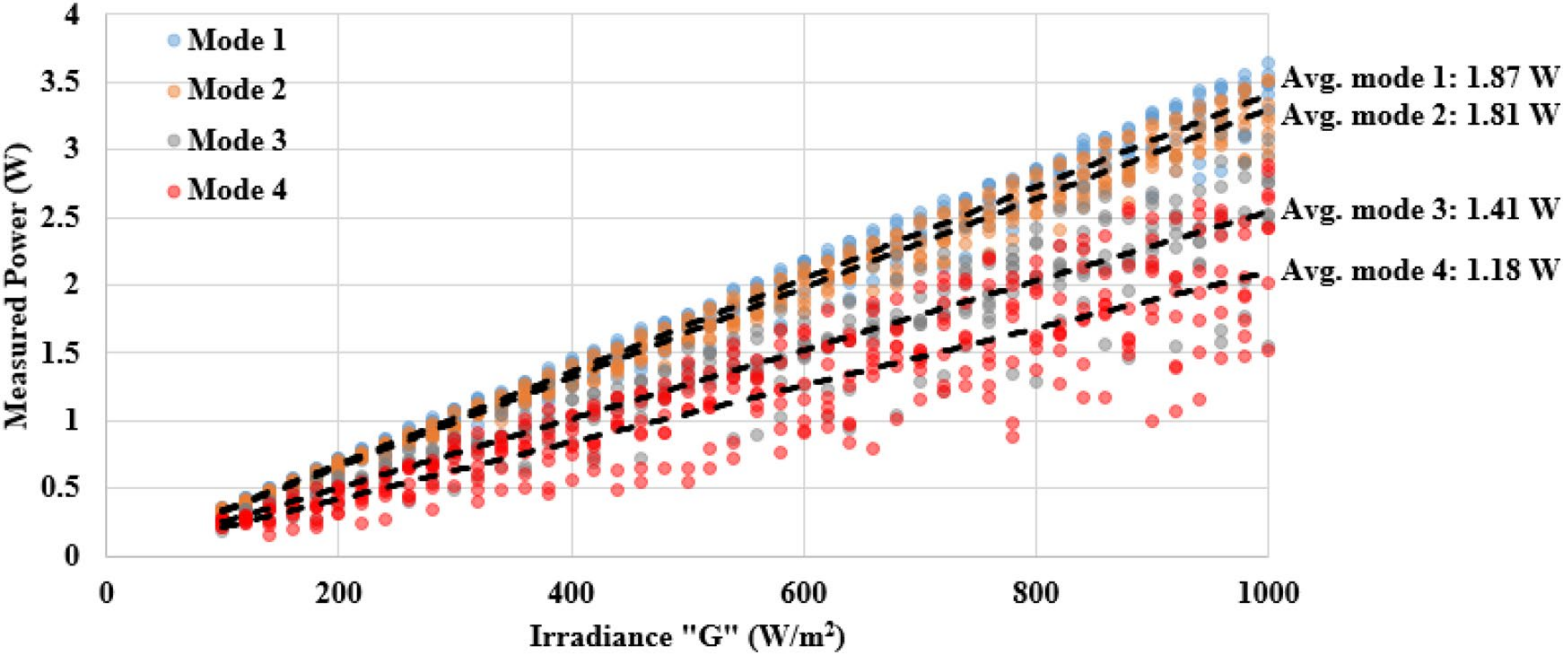
Measured power vs irradiance of the examined solar cells.

Using the solar simulator, the examined samples were subjected to illumination over the spectrum range from 100 to 1000 W/m^2^. The results of each mode are given in Fig. 6, including the linear regression. Modes 1 and 2 have a similar output power pattern, and their average measured power is 1.87 W and 1.81 W, respectively. On the other hand, a significant drop in the measured power for modes 3 and 4 is observed, averaged at 1.41 W and 1.18 W, respectively.

We show here that for modes 1 and 2, there is an insignificant change for the maximum-to-minimum output power across the range of the solar spectrum. However, for modes 3 and 4, a considerable difference in the measured power, particularly at high solar irradiance (> 500 W/m^2^).

**Figure 7:**
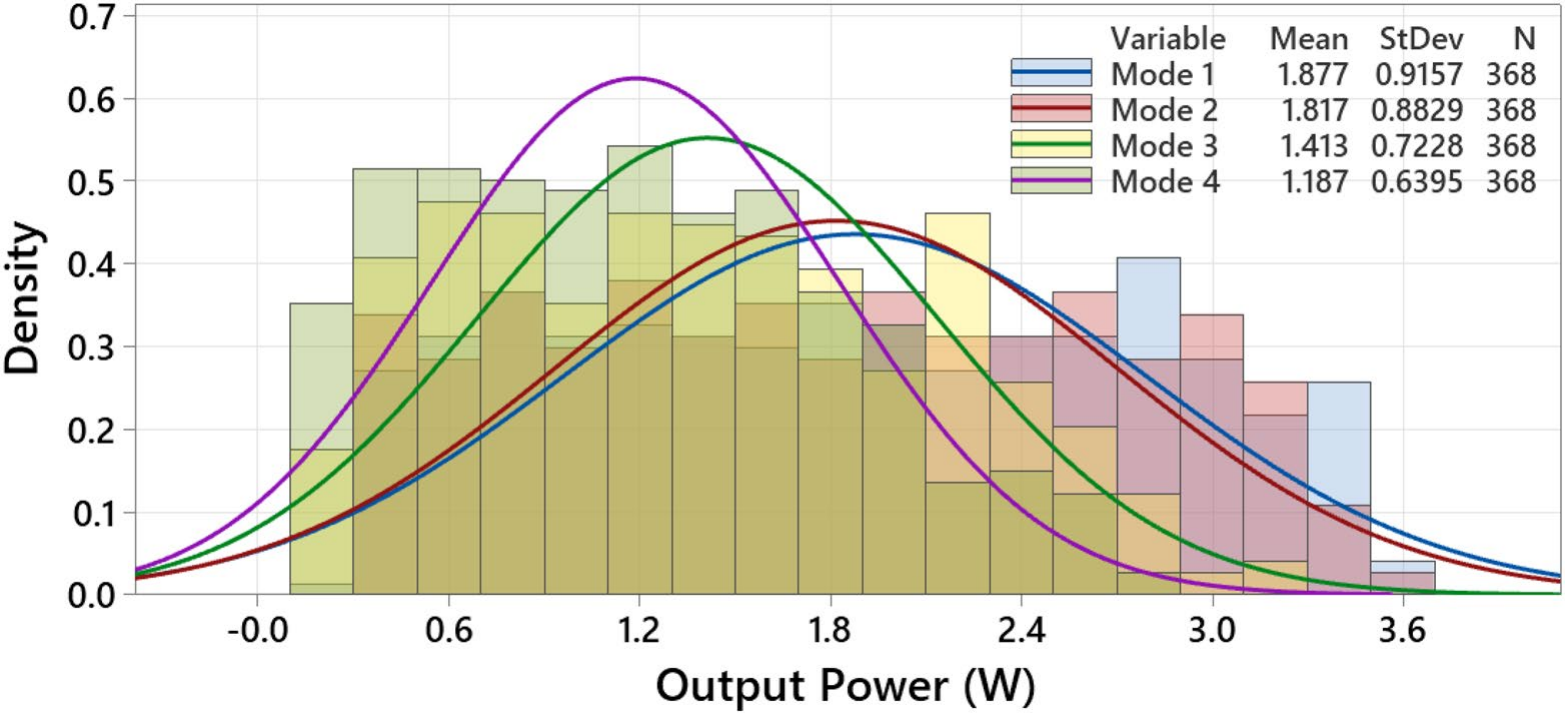
Normal distribution function applied for all measured data points.

Additionally, we summarise that the measured output power of the solar cell samples follows a normal distribution as presented in Fig. 7. However, a lower standard deviation (StDev) for modes 3 and 4 indicates that the output measure power tends to be close to the mean value. This result suggests that a significant drop in the solar cells' output power is likely to be noticed if the EL image exhibits a shaded area or breakdown regions. Finally, please note that for Fig. 7, the y-axis (density) represents the percentage of the observations in the dataset.

A correlogram is a chart of correlation statistics between different samples data; it presents the correlation between each class in the data obtained from various experiments. In our case, we have adopted the correlogram to explain the correlation between the different solar cells cracks modes. This way, we can better understand how each mode correlates with the other.

**Figure 8:**
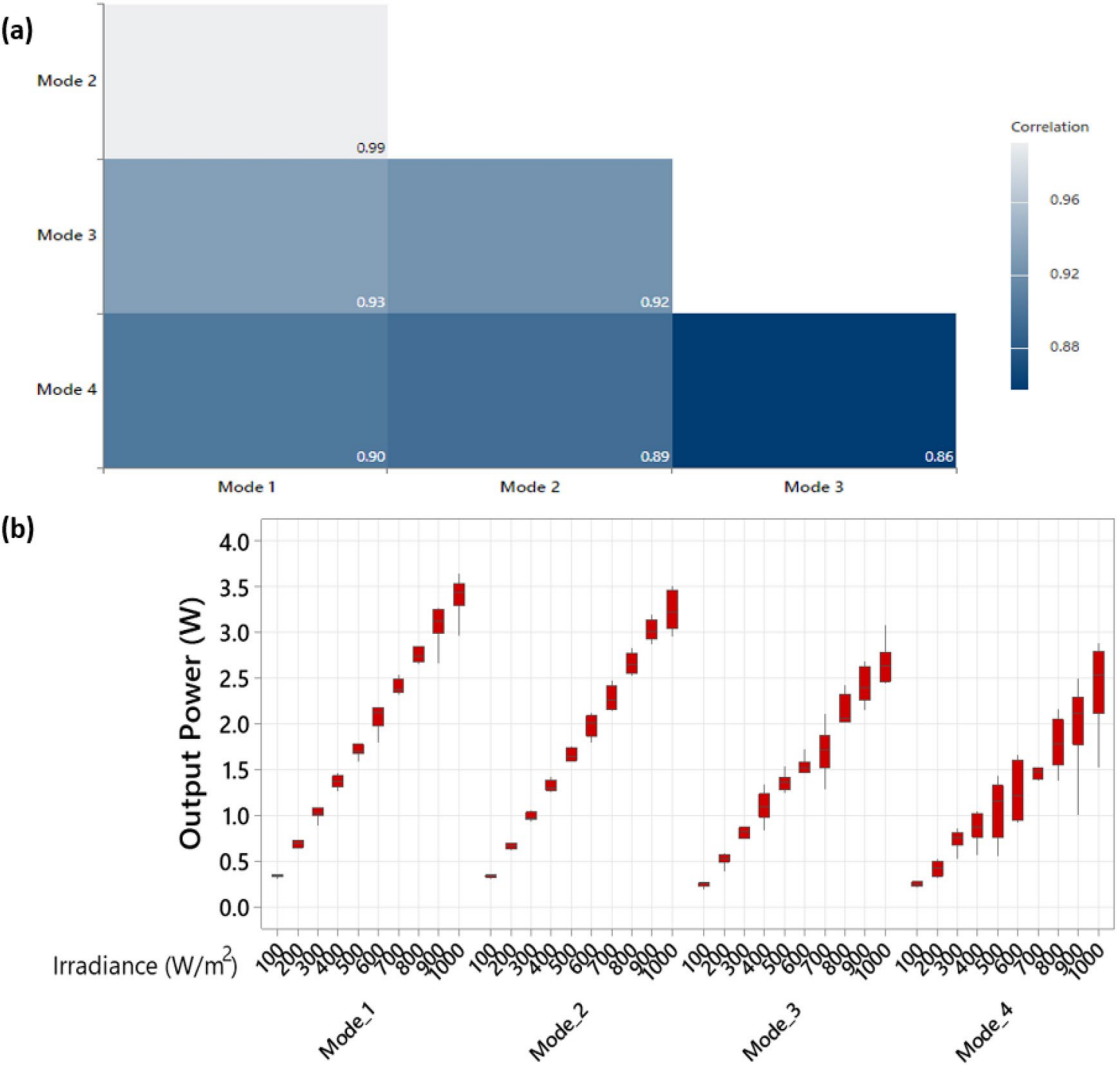
(**a**) Correlogram graph for the different solar cell crack modes investigated in this work, and (**b**) Solar irradiance vs output power of each crack mode.

Taking the measurements of the output power of each solar cell cracks mode, the correlogram is compiled and presented in Fig. 8a. In this figure, we notice that even though modes 3 and 4 looks similar in the original captured EL images, both have the least correlation, 0.86 or 86%. This outcome is found due to the nature of the cells' breakdown regions (mode 4). Therefore, they are likely to exhibit an unpredictable power loss and increase the cell's temperature with multiple levels, as small as 50$^\circ$C to 93.3$^\circ$C. On the contrary, the solar cells affected by crack mode 3 are likely to have a drop in the output power with steady fluctuations, as analysed in Fig. 8b, compared with mode 4.

Finally, and as expected, the highest correlation is marked for modes 1 and 2 at 99%. Because both crack modes exhibit the same output power losses, and they show no impact on the cell's temperature.

### Shading ratio vs hotspots development

**Figure 9:**
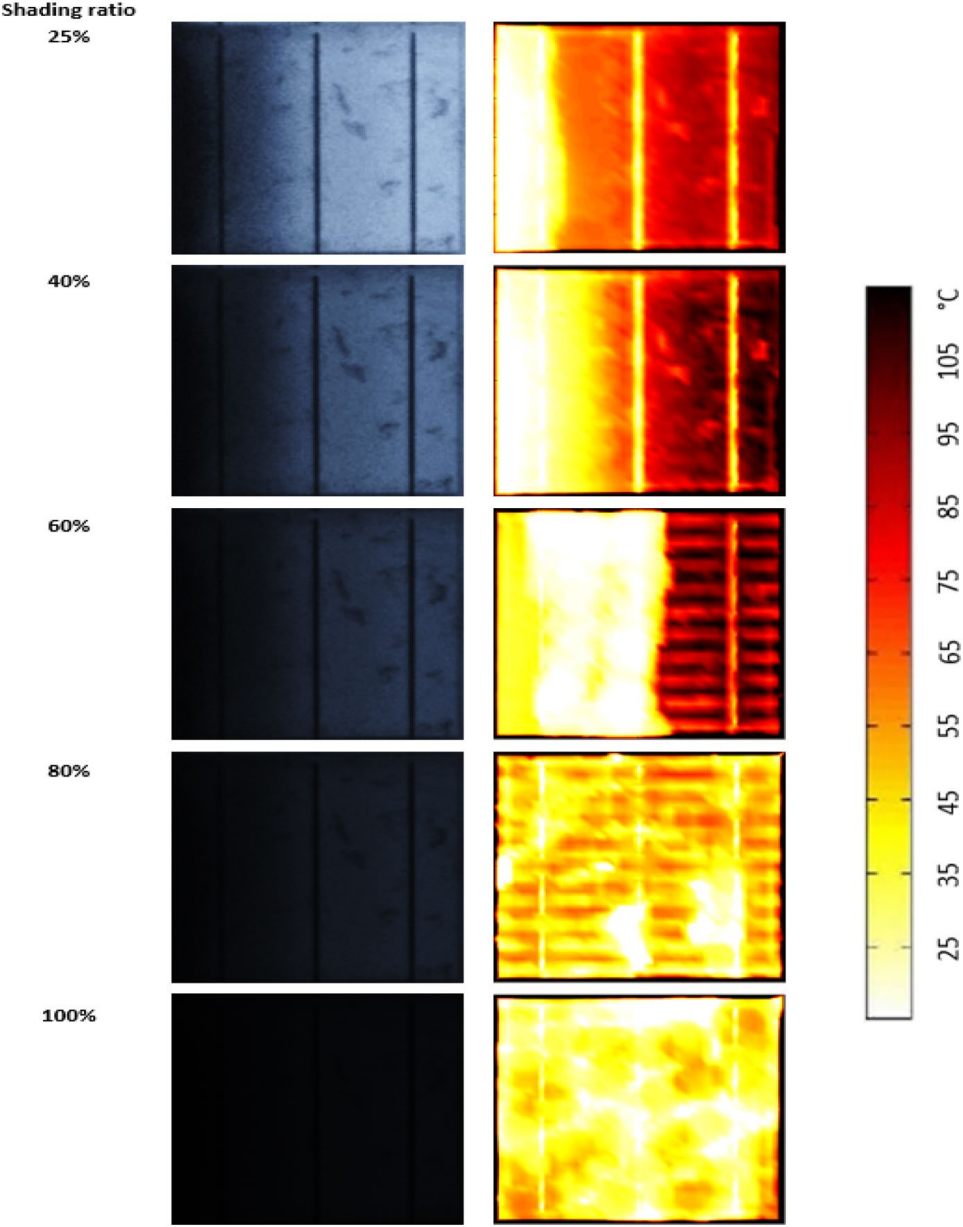
Progressive shading of a solar cell affected by mode 3 crack; the samples were subjected to illumination under STC conditions.

So far, we have evidence that solar cells affected by modes 3 and 4 are expected to have hotspots. Therefore, we have further investigated the correlation between the shade ratio in mode 3 and the presence of hotspots. The outcome of this experiment is presented in Fig. 9.

The progressive range of shade, including 25%, 40%, 60%, 80%, and 100%, is examined. If the solar cell is affected by 25% shading ratio, a maximum increase in the cell's temperature of approximately 75$^\circ$C is detected. Progressively, if the solar cell is affected by a greater shading ratio, 40% or 60%, the hotspot area has a significant increase in temperature, equivalent to 105$^\circ$C. Therefore, a hotspot is likely to develop for these three shading ratios.

For the last two shading ratios, when the solar cell is affected by 80% or 100%, no hotspot in the cell area is witnessed. The hotspot disappears due to the absence of the photo-generated current. The remaining dark current flows within the cell for insignificant cell heating and an average temperature approximately at the experimental condition, 25$^\circ$C. Thus, the worst-case scenario for the hotspot to develop occurs at the shading ratios of 40% to 60%, with an expected increase in the cell temperature from 25$^\circ$C to 105$^\circ$C.

**Figure 10:**
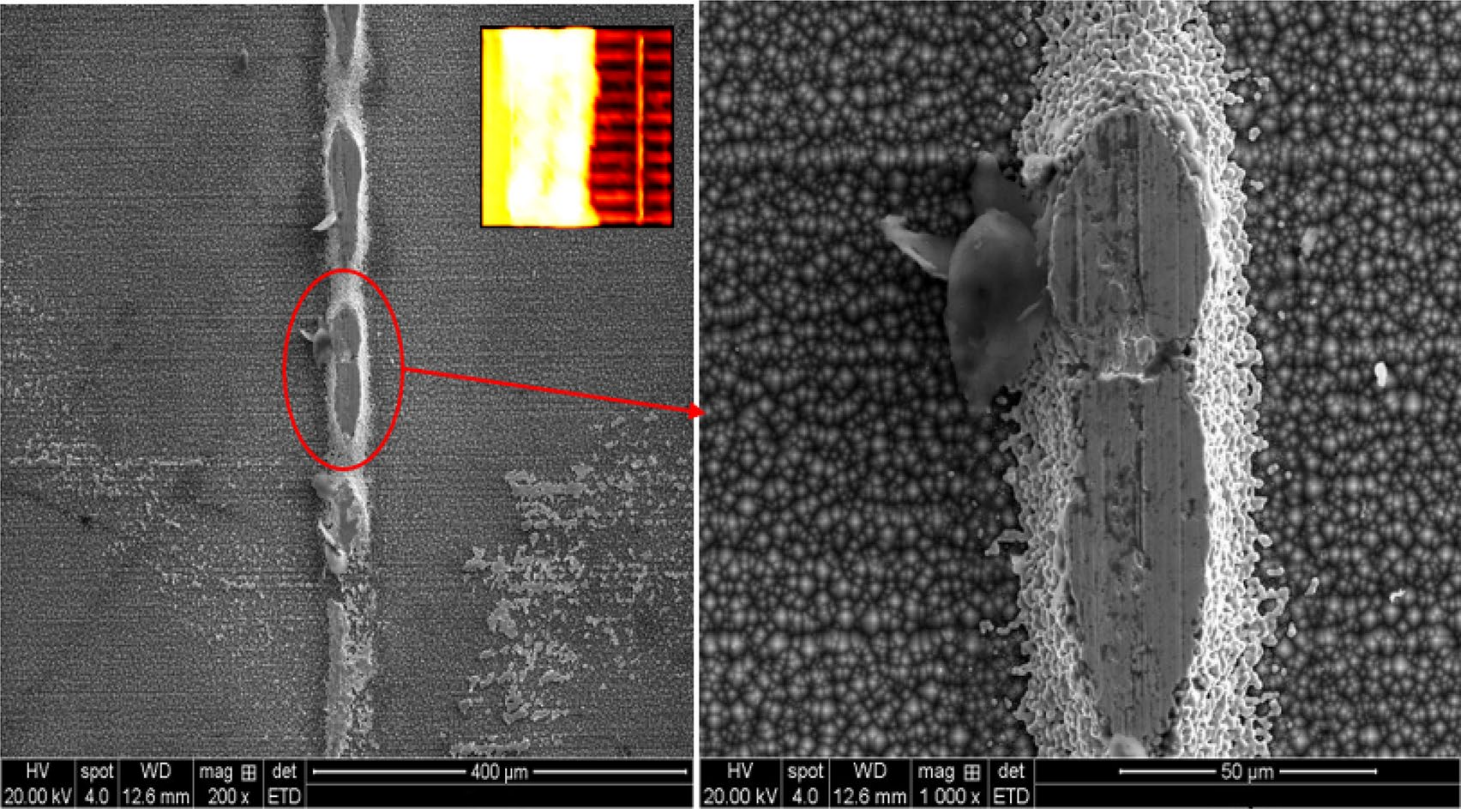
Capturing the busbar under SEM of the solar cell affected by 60% shading ratio.

Therefore, we have scanned the solar cell affected by a 60% shading ratio under SEM. This experiment was performed after the cell has been under illumination (1000 W/m^2^) for three continuous hours. The SME image of the hotspot busbar is presented in Fig. 10 under 200 and 1000 magnification. Here we can observe that the hotspot develops stress in the busbar and the surrounding area in the form of cracks. In theory, busbars are made of copper-plated silver, and silver plating is necessary to improve current conductivity.

**Figure 11:**
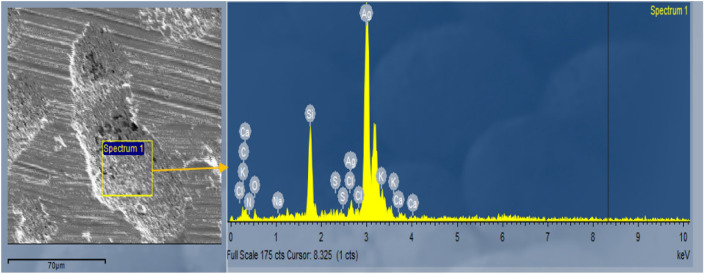
The busbar has 60% silver, 37% silicon, and 2% other material.

In this experiment, the material of the affected region of the busbar was obtained using the SEM image. According to the results shown in Fig. 11, it is recognised that the material (silicon) of the defective area of the cell is merged with silver, significantly reducing the busbar silver-plate composition, 60% silver, 37% silicon, and 2% other material. This arrangement in the solar cell is merely evidence of why the current is dropping in the non-shaded area of the cell, making this part of the cell affected by a significant increase in the temperature (hotspot).

**Figure 12:**
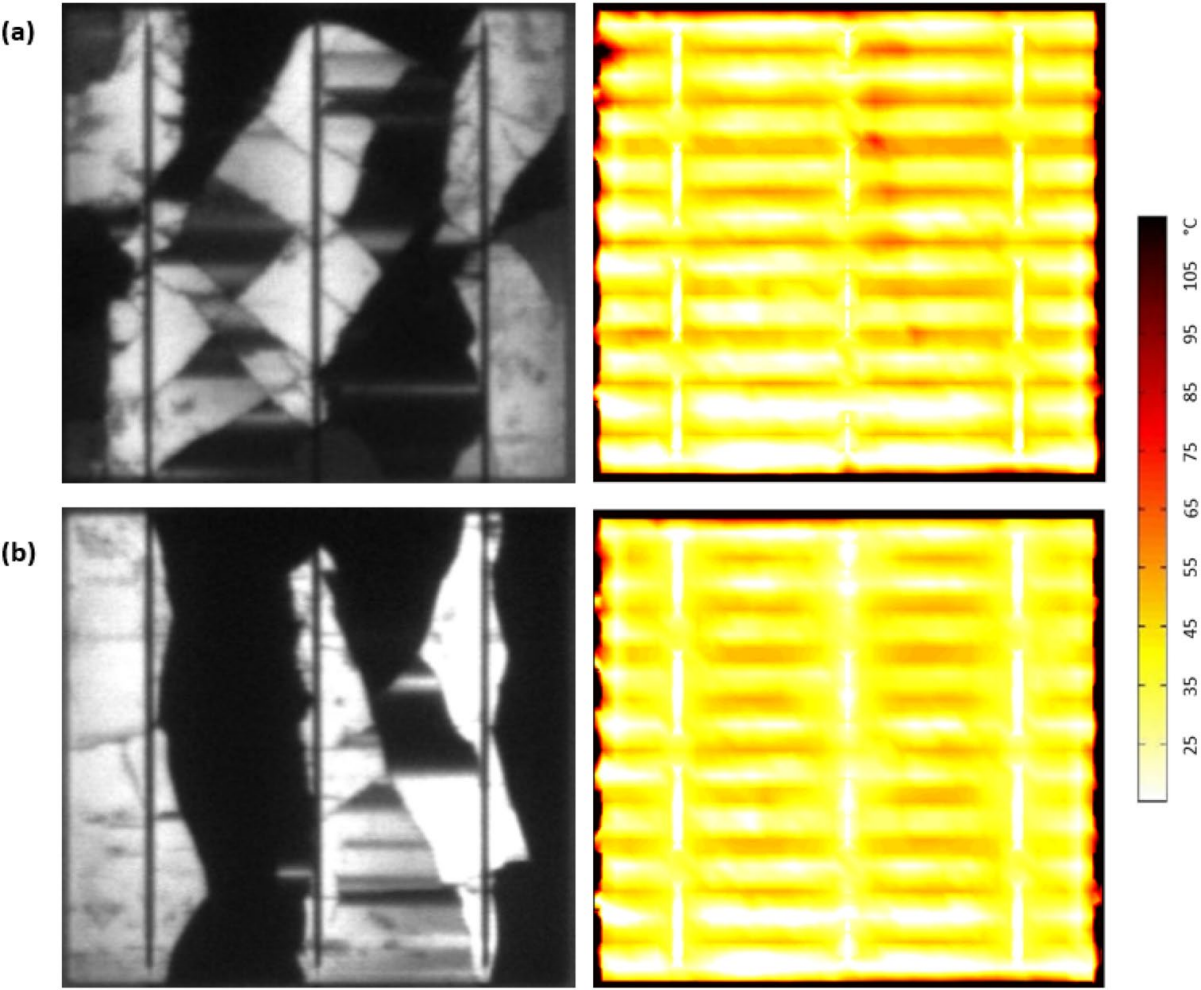
Solar cells affected by mode 4 with severe breakdown regions (**a**) sample #1, and (**b**) sample #2.

Finally, two solar cells (Fig. 12) were tested for further validation of the above-mentioned results. Both samples are affected by mode 4, with severe breakdown regions. The inactive part is approximately higher than 70%, represented by the dark black areas in the EL image. Both samples were tested under STC conditions using the solar illuminator. Because the inactive region is very high, no increase in the cell's temperature was detected. This result confirms that when the solar cell is impacted by a high ratio of shading or breakdown regions, the hotspots are unlikely to develop.

## Comparative study

To explain the significance and the new analysis presented in this study, our work has been compared, as shown in Table 1, with the most advanced recent research available in the literature^13,25,26,29^. Our work is the only one which describes the correlation between four different crack modes and the development of hotspots. All other studies have mainly considered analysing one^13,25^ or two^26^ crack modes. In contrast^29^, investigated cracks and area defects in solar cells without remarking the difference between the variations in the cracks and their correlation with the output power losses and hotspots.

**Table 1:** Comparative study of this work with previously published papers^13,25,26,29^.

Reference/year of study	Investigated solar cell cracks	Inspection Method			Correlation between crack and hotspots
		EL	thermal	SEM	
^13^/2019	Only cracks (0.5 cm) are studied	n/a	n/a	Included	n/a
^25^/2021	Only micro-cracks (5 and 10 μm) are investigated	n/a	Included	n/a	n/a
^26^/2021	Analysis based on nonuniform and uniform crack distributions	Included	n/a	Included	n/a
^29^/2021	Cracks, area defects, and finger interruptions	Included	n/a	n/a	n/a
This work/2021	Classified into four different modes: crack free, micro-crack, shade area, and breakdown	Included	Included	Included	Investigated for all crack modes

Furthermore, we have investigated the solar cell samples with different inspection techniques, including EL, thermal and SEM. On the contrary, there has not been previous research that used all three different inspection techniques to study the behaviour of cracks in solar cells.

## Conclusions

This work reports on the correlation of solar cells cracks modes and their impact on the cell's temperature, well known as hotspots. For a solar cell affected by crack modes 1 or 2, there are no hotspots detected. However, we have discovered that the solar cell is likely to have hotspots if affected by crack mode 3 or 4, with an expected increase in the temperature from 25$^\circ$C to 100$^\circ$C. Additionally, we have noticed that an increase in the shading ratio in solar cells can cause severe hotspots. With this in mind, we observed that the worst-case scenario for the hotspot to develop is at shading ratios of 40% to 60%, with a foreseen increase in the cell temperature from 25$^\circ$C to 105$^\circ$C.

## Data Availability

The datasets generated and analysed during the current study are available from the corresponding author (M.D.) on reasonable request.
